# Comparison of the retroperitoneal laparoscopic adrenalectomy versus transperitoneal laparoscopic adrenalectomy for large (≥6cm) pheochromocytomas: A single-centre retrospective study

**DOI:** 10.3389/fonc.2023.1043753

**Published:** 2023-02-22

**Authors:** Kunyang Lei, Xu Wang, Zhongsheng Yang, Yifu Liu, Ting Sun, Wenjie Xie, Ming Ma

**Affiliations:** ^1^ Department of Urology, the First Affiliated Hospital of Nanchang University, Nanchang, Jiangxi, China; ^2^ Department of Pathology, the First Affiliated Hospital of Nanchang University, Nanchang, Jiangxi, China; ^3^ Department of Urology, Ganzhou People’s Hospital, Ganzhou, Jiangxi, China

**Keywords:** pheochromocytoma, RLA, TLA, hemodynamic instability, preoperative preparation

## Abstract

**Objectives:**

To compare the efficacy and safety of retroperitoneal laparoscopic adrenalectomy (RLA) and transperitoneal laparoscopic adrenalectomy (TLA) in the treatment of large (≥6cm) adrenal pheochromocytomas.

**Methods:**

We retrospectively collected the clinical data of 130 patients with large pheochromocytoma who underwent RLA or TLA in our hospital from 2012 to 2022. The perioperative parameters and follow-up outcomes of the two groups were compared, and univariate and multivariate analyses were used to evaluate the risk factors of hemodynamic instability (HI).

**Results:**

A total of 57 patients underwent TLA and 73 underwent RLA. There was no difference in demographic characteristics such as age, sex and tumor size between the two groups. Compared with the TLA group, patients in the RLA group had shorter operation time (P<0.001) and less estimated blood loss (EBL) (P<0.001). The time to ambulation, time to oral food and time to removal of drainage of RLA group were earlier than those of TLA group. In addition, the hospital stay was shorter in the RLA group than in the TLA group. There were no differences in HI, complications, or blood pressure (BP) improvement between the two groups. The mean follow-up time was 61.4 and 65.5 months, respectively, during which no tumors recurred or metastasized. Multivariate analysis showed that elevated hormone levels and larger tumor size were independent risk factors for HI.

**Conclusions:**

Both RLA and TLA are effective treatment methods for large pheochromocytomas, but the perioperative outcomes of RLA are better than that of TLA. Our study demonstrates the superiority of RLA for the treatment of large pheochromocytomas.

## Introduction

Pheochromocytoma is a neuroendocrine tumor, most of which arise from the adrenal medulla (1). The etiology of pheochromocytoma is not known, but studies have shown that about 10% of pheochromocytomas are genetically related (2). Pheochromocytoma patients often present with hypertension, headache, arrhythmia and other clinical manifestations, which are related to the large amount of catecholamines secreted by the tumor (3). Surgical removal of the tumor is considered to be the best treatment for pheochromocytoma because it not only removes the tumor but also improves the patient’s symptoms. However, the adrenal gland is located in the posterior abdominal cavity, which makes the operation more difficult. In addition, the removal of pheochromocytoma is a very difficult task because of the abundance of blood vessels and the increased secretion of catecholamines caused by surgical procedures (4).

Laparoscopic adrenalectomy has been the standard surgical procedure for small pheochromocytomas since it was first reported in 1992 (5). Because laparoscopic surgery is less traumatic and more elaborate, this makes it have an incomparable advantage of open surgery. Studies have shown that laparoscopic adrenalectomy has a shorter operative time, lower complication rate, and faster postoperative recovery than open adrenalectomy (6, 7). Laparoscopic adrenalectomy involves transperitoneal and retroperitoneal approaches, both of which have good results. Several studies have shown that retroperitoneal laparoscopic adrenalectomy (RLA) is superior to transperitoneal laparoscopic adrenalectomy (TLA) in a number of surgical indicators (8, 9). However, these studies involved small tumors, and the difference between TLA and RLA for large pheochromocytomas (≥6cm) is unclear. The purpose of this study was to compare the perioperative and follow-up outcomes of TLA and RLA in the treatment of large pheochromocytoma, so as to provide reference for the surgical treatment of large pheochromocytoma.

## Methods

### Patients

We retrospectively collected the clinical data of pheochromocytoma patients who underwent TLA or RLA in our hospital from January 2012 to July 2022. The inclusion criteria were as follows: maximum tumor diameter ≥6cm; the postoperative specimen was pathologically confirmed as pheochromocytoma. Exclusion criteria were as follows: bilateral lesions; patients who underwent an open surgery; patients who are unable to undergo laparoscopic approach due to anesthesiologic contraindications; pheochromocytoma originating from outside the adrenal gland; clinical data were incomplete. All the patients signed an informed consent form. Our study was approved by the Ethics Committee of the First Affiliated Hospital of Nanchang University.

### Preoperative preparation

All patients underwent CT scan and ultrasound before operation. Baseline hormone levels were assessed in all patients before surgery. The evaluation content included cortisol, dopamine, norepinephrine, epinephrine, adrenocorticotropic hormone, and serum potassium. The levels of 24-h urinary dopamine, norepinephrine, epinephrine and vanillylmandelic acid were also evaluated. All patients were treated with oral α-receptor blockers for at least 2 weeks before surgery. Patients with tachycardia should also be treated with β-receptor blockers. All patients received intravenous fluids to expand their blood volume 3-5 days before surgery. Patients can undergo surgery when they meet the following criteria: blood pressure (BP) <140/90 mmHg, heart rate <100/min, haematocrit <45%, and warm fingertips.

### Baseline data and outcome indicators

Data to be evaluated include patient demographics and perioperative outcomes. Demographic characteristics included gender, age, body mass index (BMI), hypertension, diabetes, arrhythmia, ASA score, hormone levels, tumor side, tumor size and CT findings. Perioperative indicators included operation Time, pneumoperitoneum time, estimated blood loss (EBL), hemodynamic instability (HI), time to ambulation, time to oral food, time to removal of drainage, postoperative hospital stay, complications, and BP improvement rate.

The ASA score was used to evaluate the patient’s tolerance to anesthesia. Complications were classified according to the Clavien-Dindo classification (10). HI was defined as intraoperative BP >180 mmHg or mean arterial pressure <60 mmHg (11). BP improvement was defined as a decrease in BP after surgery or a decrease in the dose or type of antihypertensive medication taken (12).

### Surgical technique

The choice of surgical method is based on the wishes of the patient and the preferences of the surgeon. All the operations were performed by six senior surgeons from our department. The specific steps of TLA and RLA have been explained in detail in previous studies (9).

### Follow-up

Follow-up was performed every 3 months during the first year after surgery and every 6 months thereafter. Follow-up included routine physical examination, hematology, hormone levels, and abdominal and chest imaging. For patients who could not come to our hospital for review, we all evaluated their health status by telephone.

### Statistical analysis

All data in this study were statistically analyzed using IBM SPSS26.0. Continuous variables conforming to normal distribution were expressed as mean and standard deviation (SD), and comparisons between groups were performed using independent sample t-test. Continuous variables that did not conform to normal distribution were expressed as median and interquartile range (IQR), and comparison between groups was performed using the Mann-Whitney U test. Categorical variables were expressed as percentages, and comparisons between groups were performed using Pearson’s chi-square test. Univariate and multivariate logistic regression models were used to evaluate the risk factors for HI. A P value of <0.05 was considered statistically significant.

## Results

The preoperative characteristics of patients are summarized in **Table 1**. Fifty-seven patients underwent TLA and 73 underwent RLA. Overall, there were no differences in gender, age, BMI, Hypertension, Diabetes, Arrhythmia, ASA score ≥3, hormone levels, tumor side, tumor size and CT findings between the two groups.

**Table 1:** Preoperative characteristics of patients.

Variables	TLA (n=57)	RLA (n=73)	P value
Age, mean (SD), year	45.6 (13.6)	49.1 (12.6)	0.133
Gender, n (%)			0.190
Male Female	30 (52.6) 27 (47.4)	30 (41.1) 43 (58.9)	
BMI, median (IQR), kg/m^2^	20.8 (20.1-21.5)	21.0 (20.0-21.8)	0.250
Hypertension, n (%)	33 (57.9)	39 (53.4)	0.611
Diabetes, n (%)	16 (28.1)	19 (26.0)	0.794
Arrhythmia, n (%)	5 (8.8)	7 (9.6)	0.873
ASA score ≥ 3, n (%)	7 (12.3)	7 (9.6)	0.623
Elevated hormone levels, n (%)	22 (38.6)	31 (42.5)	0.656
Tumor side, n (%)			0.948
Left Right	27 (47.4) 30 (52.6)	35 (47.9) 38 (52.1)	
Tumor size, median (IQR), cm	7.5 (6.0-9.0)	7.2 (6.5-9.0)	0.902
CT findings, n (%)			
Calcification Cystic degeneration Irregular tumor shape	5 (8.8) 25 (43.9) 18 (31.6)	8 (10.9) 33 (45.2) 20 (27.4)	0.680 0.878 0.603

BMI, Body mass index; SD, Standard deviation; IQR, interquartile range; TLA, transperitoneal laparoscopic adrenalectomy; RLA, retroperitoneal laparoscopic adrenalectomy.

In terms of perioperative outcomes, the operative time was significantly shorter in the RLA group than in the TLA group (P=0.001). There was no difference in pneumoperitoneum time between the two groups. The RLA group had significantly less EBL than the TLA group (P <0.001). In addition, the time to ambulation, time to oral food and time to removal of drainage of the RLA group were shorter than those of the TLA group (all P <0.001). Compared with the TLA group, the postoperative hospital stay was significantly shorter in the RLA group (P<0.001). There were no significant differences in the incidence of complications or the rate of BP improvement between the two groups. The mean follow-up time was 61.4 (29.4) months in the TLA group and 65.5 (31.7) months in the RLA group. There was no tumor recurrence or metastasis in the two groups during the follow-up (**Table 2**).

**Table 2:** Perioperative outcomes.

Variables	TLA (n=57)	RLA (n=73)	P value
Operative time, median (IQR), min	170.0 (155.0-182.5)	156.0 (144.0-165.0)	0.001
Pneumoperitoneum time, median (IQR), min	138.0 (125.0-152.0)	125.0 (115.0-135.0)	0.908
EBL, median (IQR), min	180.0 (140.0-270.0)	100.0 (120.0-150.0)	<0.001
HI, n (%)	28 (49.1)	34 (46.6)	0.773
Conversion to open, n (%)	3 (5.2)	5 (6.8)	0.708
Transfusion, n (%)	4 (7.0)	3 (4.1)	0.466
Time to ambulation, median (IQR), day	2.0 (1.0-2.0)	1.0 (1.0-2.0)	0.001
Time to oral food, median (IQR), day	2.0 (2.0-3.0)	1.0 (1.0-2.0)	<0.001
Time to removal of drainage, median (IQR), day	5.0 (4.0-5.0)	3.0 (3.0-4.0)	<0.001
Postoperative hospital stay, median (IQR), day	7.0 (7.0-8.0)	6.0 (5.0-6.0)	<0.001
Total complications Clavein-Dindo grade 1-2, n (%) Clavein-Dindo grade 3-4, n (%)	27 (47.3) 17 (29.8) 10 (17.5)	28 (38.3) 22 (30.1) 6 (8.2)	0.302 0.969 0.108
BP improvement, n (%)	29 (87.9)	34 (87.2)	0.929
Duration of follow-up, mean (SD), month	61.4 (29.4)	65.5 (31.7)	0.460

SD, Standard deviation; IQR, interquartile range; TLA, transperitoneal laparoscopic adrenalectomy; RLA, retroperitoneal laparoscopic adrenalectomy; EBL, estimated blood loss; HI, hemodynamic instability; BP, blood pressure.

To assess the risk factors for intraoperative HI, logistic regression analysis was performed (**Table 3**). Univariate analysis showed that elevated hormone levels, larger tumor size, longer operation time and pneumoperitoneum time, and more EBL were risk factors for HI (all P <0.001). Multivariate analysis showed that elevated hormone levels and larger tumor size were independent risk factors for HI (P <0.001, P=0.003 respectively).

**Table 3:** Univariable and multivariable analysis for HI.

Variables	Univariable analysis			Multivariable analysis		
	OR	95% CI	P value	OR	95% CI	P value
Age	1.000	0.974-1.027	0.977	1.028	0.959-1.102	0.434
Gender	1.187	0.595-2.369	0.626	0.407	0.120-1.374	0.147
BMI	1.013	0.973-1.055	0.520	1.027	0.973-1.058	0.337
Hypertension	0.959	0.480-1.916	0.905	1.098	0.251-4.806	0.901
Diabetes	1.049	0.483-2.279	0.903	0.880	0.145-5.355	0.890
Arrhythmia	0.764	0.230-2.546	0.662	0.671	0.103-4.381	0.677
ASA score ≥ 3	1.109	0.366-3.363	0.855	1.389	0.112-17.290	0.798
Elevated hormone levels	0.112	0.048-0.258	<0.001	0.035	0.007-0.177	<0.001
Tumor side	1.194	0.599-2.379	0.982	1.763	0.548-5.674	0.341
Tumor size	0.312	0.209-0.467	<0.001	0.222	0.083-0.595	0.003
Calcification	0.934	0.296-2.946	0.907	5.697	0.549-59.102	0.145
Cystic degeneration	0.813	0.406-1.626	0.557	0.515	0.133-2.000	0.338
Irregular tumor shape	1.537	0.718-3.286	0.268	2.064	0.592-7.190	0.255
TLA vs. RLA	1.108	0.553-2.216	0.773	0.478	0.009-24.480	0.713
Operative time	0.951	0.930-0.972	<0.001	1.004	0.770-1.308	0.978
Pneumoperitoneum time	0.936	0.909-0.964	<0.001	1.007	0.733-1.383	0.966
EBL	0.981	0.974-0.989	<0.001	1.000	0.983-1.018	0.973

BMI, Body mass index; TLA, transperitoneal laparoscopic adrenalectomy; RLA, retroperitoneal laparoscopic adrenalectomy; EBL, estimated blood loss; OR, odds ratio; CI, confidence interval.

## Discussion

The surgical treatment of adrenal pheochromocytoma is a very challenging task because of the special anatomical location of the adrenal gland, which makes it difficult for the surgeon to operate. In the past, removing the adrenal gland often required a large incision, even for small adrenal tumors. In addition, pheochromocytomas are very rich in blood vessels, often leading to severe bleeding during surgery. Moreover, because pheochromocytomas are neuroendocrine tumors that secrete large amounts of catecholamines, the surgeon’s compression of the tumor during surgical resection can cause severe HI (13, 14). The use of laparoscopy has solved all of these problems. First, the surgeon can remove the tumor without having to make a large incision. Secondly, the magnification function of laparoscopy enables the surgeon to better identify the blood vessels of the tumor, thus better hemostasis. In addition, compared with open surgery, laparoscopic resection of pheochromocytoma is performed more gently, thus avoiding the occurrence of HI (15). Robotic surgical systems overcome the limitations of laparoscopic surgery, such as two-dimensional imaging and surgeon discomfort. Robot-assisted laparoscopic adrenalectomy involves less EBL and faster postoperative recovery than laparoscopic adrenalectomy (16, 17). For large adrenal pheochromocytomas, many scholars believe that open surgery should be used, because they believe that these tumors tend to be malignant and that open surgery can facilitate a more complete resection of the tumor. In addition, the limited surgical space in the posterior abdominal cavity is not conducive to laparoscopic resection of large pheochromocytomas. However, several studies have shown that laparoscopic adrenalectomy is more effective and safer than open adrenalectomy for large adrenal pheochromocytoma (18, 19). The above findings provide a good basis for laparoscopic adrenalectomy in the treatment of large pheochromocytoma. Therefore, this study compared two approaches of laparoscopic adrenalectomy, namely TLA and RLA, in order to clarify the advantages and disadvantages of the two approaches, so as to provide reference for the surgical treatment of large pheochromocytoma.

The transperitoneal approach was the first approach to be used, and it was once the most popular approach. The transperitoneal approach offers more space for manoeuvre, a better vision, and a more defined anatomical structure, allowing the adrenal gland to be easily located. However, this approach inevitably affects the intestine and may result in intestinal adhesions. In addition, vital organs such as the liver and pancreas are also vulnerable to damage, which can lead to serious complications (20). The advantage of the retroperitoneal approach is that it avoids affecting organs such as the intestine, so patients recover more quickly after surgery. In addition, because this approach goes directly into the posterior abdominal cavity, where the adrenal gland is located, surgical time is saved. However, due to the limited space of the posterior abdominal cavity, the surgeon’s manipulation may be limited during the removal of large adrenal tumors (21). A retrospective study by Li et al. was the first to compare the perioperative outcomes of RLA and TLA for pheochromocytoma (8). Their study showed that the RLA group was superior to the TLA group in terms of operation time, EBL, postoperative hospital stay, and complications. However, their study only included patients with tumors smaller than 6cm, making it difficult to uncover the drawbacks of RLA. Shiraishi et al. compared the perioperative results of RLA and TLA in the treatment of pheochromocytoma larger than 5cm, and their findings also confirmed the superiority of RLA in terms of operation time, pneumoperitoneum time, and EBL (22). However, their study included only 22 patients, so the reliability of their findings may be limited. In the present study, the RLA group outperformed the TLA group on a number of perioperative indicators, such as shorter postoperative hospital stays in the RLA group than in the TLA group, which is similar to what has been reported. These advantages of RLA are related to the small impact on the intestine and other organs in the abdominal cavity, which makes patients less traumatic and recover faster after surgery. Although the limited space in the posterior abdominal cavity may prolong the operation time when removing large tumors, this did not occur in our study. This is because our center has been using this technology for a long time, so it has developed a skilled operation process. It can be seen that for experienced surgeons, the operation time will not be significantly prolonged even if the operating space is limited.

HI is one of the biggest challenges that surgeons face when removing pheochromocytoma because it can be life-threatening. Studies have shown that the incidence of HI during adrenalectomy ranges from 17 to 83%, which is generally consistent with our results (23). The occurrence of HI is related in tumor traction, therefore, gentle manipulation is the key to avoid HI. In addition, preoperative administration of α-receptor blockers and early adrenal central vein ligation prevented adverse cardiovascular and cerebrovascular events, although they did not reduce the frequency of HI (24). Once HI occurs, it is very important to use medicine promptly to keep the patient’s vital signs stable. Therefore, an experienced anesthesiologist is one of the keys to a successful pheochromocytoma resection (25). Our findings suggest that elevated hormone levels and larger tumor size were independent risk factors for HI during pheochromocytoma resection. According to previous studies, the development of HI is related to the size of the tumor, because the larger the tumor, the higher the level of catecholamines (26, 27). In addition, other studies have shown that the preoperative hormone level of patients is also one of the key factors affecting the occurrence of intraoperative HI, which is consistent with our findings (28, 29). Therefore, for large adrenal pheochromocytoma, surgeons should prevent the occurrence of HI to ensure patient safety.

Our study has several limitations. First, this study is a retrospective study. Secondly, the choice of surgical methods is based on the surgeon’s preference, which may lead to selection bias. Finally, although this is the largest study involving large pheochromocytoma patients to date, the number of cases is still limited, so we look forward to large randomized controlled trials in the future.

## Conclusion

Both RLA and TLA are effective treatment methods for large pheochromocytoma, but the perioperative outcome of RLA is better than that of TLA. Larger tumor size and elevated hormone levels are independent risk factors for HI. Perfect preoperative preparation and gentle intraoperative operation are the key to prevent HI. Our study demonstrates the feasibility and superiority of RLA in the treatment of large adrenal pheochromocytoma.

## Data availability statement

The raw data supporting the conclusions of this article will be made available by the authors, without undue reservation.

## Ethics Statement

All the patients signed an informed consent form. Our study was approved by the Ethics Committee of the First Affiliated Hospital of Nanchang University.

## Author contributions

KL and XW conceived the study. YL collected the data. WX, TS and ZY re-examined the data. KL and XW analyzed the data. KL, XW and ZY wrote the manuscript. MM and ZY reviewed the manuscript. ZY revised the manuscript. All authors contributed to the article and approved the submitted version.
